# Supplementary material for: Pulmonary Conventional Type 1 Langerin-Expressing Dendritic Cells Play a Role in Impairing Early Protective Immune Response against Cryptococcus neoformans Infection in Mice
Source: J Fungi (Basel). 2022 Jul 28;8(8):792. doi: 10.3390/jof8080792 (PMC9410147; doi:10.3390/jof8080792)
Supplement: Supplementary file 1 [file jof-08-00792-s001.zip › jof-1765540-supplementary.pdf]

Supplementary Figure S1, related to Figure 2

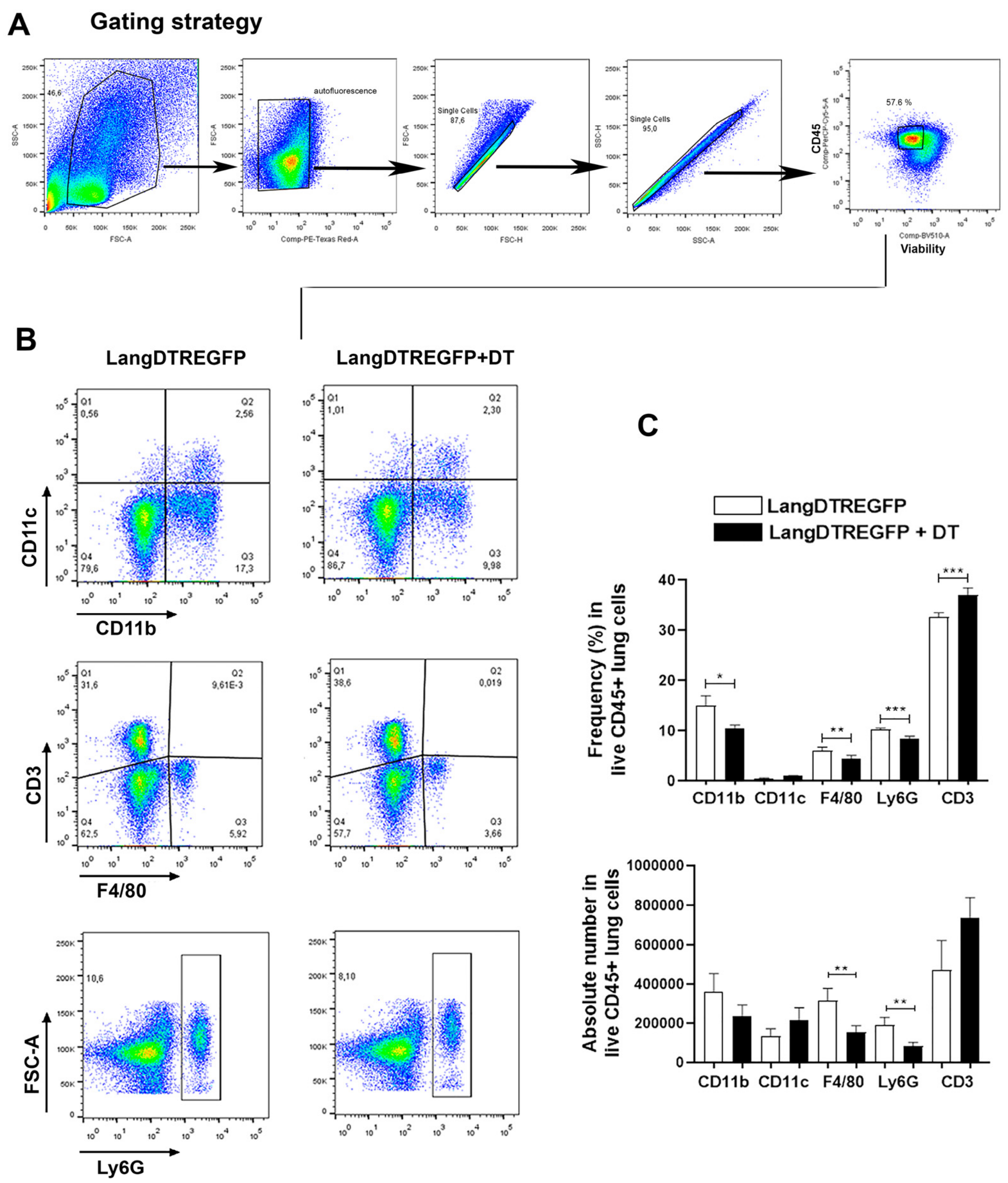

Figure S1. Flow cytometry analysis of lung cell suspensions from *C. neoformans*-infected *LangDTREGFP* mice (7 dpi). (**A**) Gating strategy used to identify immune cell subsets in lung cell suspensions. (**B**) Representative dot plots and (**C**) bar graphs showing the frequency and absolute number of CD11c, CD11b, CD3, F4/80 and Ly6G on live CD45 positive cells. Each bar shows mean + SEM. Statistical differences were evaluated by unpaired t-test. * $p < 0.02$; ** $p < 0.05$; *** $p < 0.01$.
